# Palliative Management of Malignant Esophago-Tracheobronchial Fistula Using Covered Self-Expandable Metallic Stents: A Single-Center Case Series

**DOI:** 10.1177/26892820261428670

**Published:** 2026-03-17

**Authors:** Keisuke Eguchi, Takahiro Nakajima, Takeshi Terashima

**Affiliations:** 1 Department of General Thoracic Surgery, Tokyo Dental College Ichikawa General Hospital, Chiba, Japan.; 2 Respiratory Medicine, Tokyo Dental College Ichikawa General Hospital, Chiba, Japan.

**Keywords:** covered self-expandable metallic stent, double stenting, endoscopic intervention, esophago-tracheobronchial fistula, palliative management, thoracic malignancy

## Abstract

**Background:**

Malignant esophago-tracheobronchial fistula (ETBF) is a devastating complication of thoracic malignancies, especially advanced esophageal cancer. Covered self-expandable metallic stents (CSMSs) have emerged as a minimally invasive palliative option.

**Methods:**

Between May 2015 and June 2021, seven patients with malignant ETBF (five men and two women; mean age 63 ± 5.8 years) underwent CSMS placement at our institution. Primary diseases included five esophageal cancers and two metastatic mediastinal carcinomas. Clinical characteristics, technical success, complications, and outcomes were retrospectively reviewed.

**Results:**

No intraoperative mortality occurred. All patients experienced improvement in dysphagia and/or airway aspiration, allowing resumption of oral intake, which was maintained for a median of 62 days (range, 10–124 days). Median post-stenting survival was 104 days (range, 14–250 days).

**Conclusion:**

CSMS placement is a safe and effective palliative treatment for malignant ETBF, preventing aspiration pneumonia and restoring oral intake, thereby supporting home-based care.

## Introduction

Patients with mediastinal malignancies, including advanced esophageal cancer, may develop tracheobronchial fistulas and/or stenosis during the course of disease progression. Martini et al. reported that esophago-tracheobronchial fistula (ETBF) formation is a particularly serious complication, occurring in 14.75% of patients with tracheal cancer, 4.94% with esophageal cancer, and 0.16% with lung cancer.^1^ ETBF may also occur as a result of chemoradiotherapy for esophageal cancer with tracheobronchial invasion, with an incidence of 20–30%.^2^ Dysphagia significantly impairs quality of life (QOL), affecting daily activities, social participation, nutritional intake, and multiple patient-reported domains, including symptoms, eating desire, burden, food selection, communication, and mental health.^3^ In esophago-tracheobronchial fistula (ETBF)-related dysphagia, fear is particularly heightened regarding aspiration pneumonia, further contributing to anxiety, depression, and burnout.^4^

While several surgical approaches to managing malignant ETBFs have been reported, the operative mortality is prohibitively high.^5^ Although closure of the ETBF may improve patients’ QOL, most are in a terminal condition with a life expectancy of less than three months,^1^ and highly invasive procedures should be avoided whenever possible. A stent used in the treatment of tracheobronchial or esophageal disorders is a hollow prosthesis that is endoscopically inserted and deployed to maintain patency of the lumen in areas affected by stenosis or malacia or to occlude fistulous lesions. Various types of stents have been developed to date, while self-expandable metallic stents are composed of a metallic mesh that expands the lumen by its intrinsic elasticity. Covered self-expandable metallic stents (CSMSs) are an improved form of self-expandable metallic stents, in which the metallic mesh is covered with a silicone membrane to prevent occlusion caused by tumor ingrowth or granulation tissue through the mesh. CSMSs can be placed in both the esophagus and tracheobronchus, and double stenting is performed to ensure reliable ETBF closure. They are technically simple to deploy and effective for fistula occlusion at either site;^6,7^ therefore, we focused on applying CSMSs to the treatment of ETBF, taking advantage of their unique properties.

We report the outcomes of palliative treatment of ETBFs in patients with malignancies by placement of a CSMS.

## Patients and Methods

### Setting and patients

Seven patients with malignant ETBF underwent stenting at Tokyo Dental College Ichikawa General Hospital (Chiba, Japan) between May 2015 and June 2021 (Table 1).

**Table 1.** Patient Characteristics and Treatments for Esophago-Tracheobronchial Fistula

case	Sex	Age	Primary disease	Treatment before stenting	Reason for impaired oral intake	TB tumor stenosis	Fistula observed by bronchoscopy	Location of TB lesion	TB stent size (mm)	Es tumor stenosis	Es stenting
1	F	66	MMC	CT	Liquid choking	+	Yes	Left MB	12 × 40	+	Done
2	M	68	EC	None	Aspiration pneumonia	−	Yes	Left MB	14 × 60	+	Done
3	M	68	EC	CRT	Chest pain (swallowing)	+	No	Left MB	14 × 60	−	Done
4	F	56	EC	None	Obstructive sensation (Swallowing)	+	Yes	Lower trachea	20 × 80	+	Done
5	M	69	EC	RT	Obstructive sensation (Swallowing)	−	Yes	Lower trachea	12 × 40	−	—
6	M	60	EC	None	Liquid choking	+	No	Mid-trachea	18 × 60	+	Done
7	M	56	MMC	CRT	Liquid choking	−	Yes	Lower trachea	20 × 40	−	—

M, male; F, female; MMC, metastatic mediastinal carcinoma of unknown origin; EC, esophageal cancer; CT, chemotherapy; CRT, chemoradiotherapy; RT, radiotherapy; TB, tracheobronchial; BS, bronchoscopy; MB, main bronchus; Es, esophageal.

There were five men and two women, aged 56–69 years (mean, 63 years). Primary disease was esophageal cancer in five (stage III in four [cases 2, 3, 4, and 5], stage IVA in one [case 6]) and metastatic mediastinal carcinoma of unknown origin in two (cases 1 and 7). Presenting symptoms included dysphagia, cough, fever, and recurrent pneumonia. Reasons for impaired oral intake included choking during water ingestion (cases 1, 6, and 7), aspiration pneumonia (case 2), chest pain on swallowing (case 3), and sensation of obstruction during swallowing (cases 4 and 5).

**FIG. 1.**
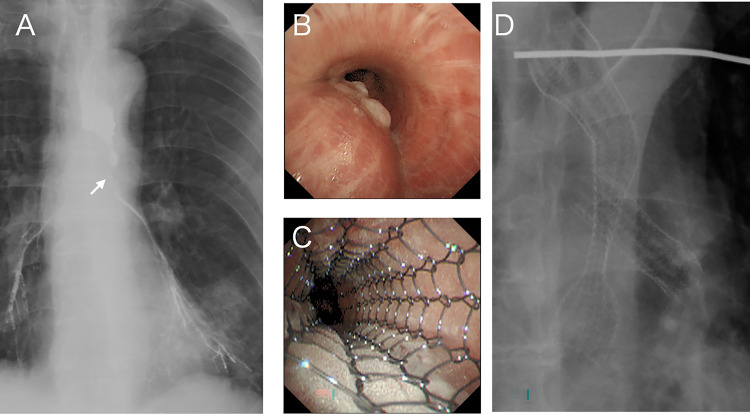
Radiological and endoscopic findings of case 3: Fistulography showing the esophago-bronchial fistula (arrow, A); bronchoscopic image showing tumor invasion in the left main bronchus **(B)** and the inserted bronchial stent **(C)**; A chest X-ray showing the “double stenting, “namely, both bronchial and esophageal stenting **(D)**.

ETBF was confirmed by bronchoscopy and/or fistulography (Fig. 1A). In two patients (cases 3 and 6), the fistula could not be confirmed directly by bronchoscopy (Fig. 1B). Fistula sites were: left main bronchus (cases 1, 2, and 3), mid-trachea (case 6), and lower trachea (cases 4, 5, and 7). Four patients (cases 1, 3, 4, and 6) also had tracheobronchial stenosis. Prior therapies included chemotherapy (case 1), radiotherapy (case 5), chemoradiotherapy (cases 3 and 7), or none (cases 2, 4, and 6). None were candidates for radical surgery.

### Procedure overview

All patients underwent CSMS placement under topical pharyngeal anesthesia (2% lidocaine) with intravenous diazepam and/or pethidine hydrochloride sedation. Airway stents^8–10^ used were *Ultraflex™ (Boston Scientific,* Fig. 1C). Esophageal stents^11,12^ included *Ultraflex™* (cases 1, 2, 3, and 6) and *HANAROSTENT™* (case 4). The *HANAROSTENT™* is characterized by complete coverage of the metallic mesh with silicone, representing an improvement over the *Ultraflex*™ stent, in which the mesh near both ends remains uncovered. Endoscopic procedures were performed using a flexible video bronchoscope or gastroscope system (Olympus, Tokyo, Japan). Double stenting was performed in five patients (cases 1, 2, 3, 4, and 6; Fig. 1D).

## Results

**FIG. 2.**
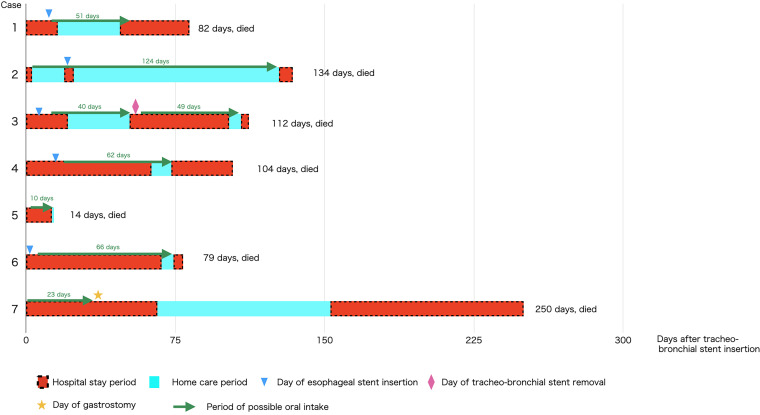
A swimming plot of the clinical courses of seven cases with esophago-tracheobronchial fistula reported herein.

All procedures were technically successful with no intraoperative mortality. The clinical courses of the seven patients are shown in Figure 2. All patients experienced improvement in airway aspiration and/or dysphagia following stent placement, enabling them to resume oral intake, which lasted a median of 62 days (range, 10–124 days). Median post-stenting survival was 104 days (range, 14–250 days). The reasons for discontinuation of resumed oral intake were all attributable to tumor progression, including loss of appetite (cases 4 and 6), sudden death at home (case 5), respiratory distress due to aspiration pneumonia (cases 2 and 3), and worsening esophageal stenosis (cases 1 and 7).

### Representative cases

Case 3: The patient was readmitted with obstructive pneumonia caused by stent migration and sputum retention, necessitating removal of the airway stent after 46 days; thereafter, the esophageal stent alone successfully maintained fistula closure.

Case 5: Despite effective radiotherapy, ETBF developed; an airway stent enabled temporary oral intake; however, the patient died shortly after discharge.

Case 7: Only a bronchial stent was placed; oral intake was possible for 23 days, but gastrostomy was required for nutrition.

All patients were discharged at least temporarily, with a median home stay of 31 days (range, 1–119 days). Ultimately, all died from progression of their primary malignancy. In case 4, treated with *HANAROSTENT*™, outcomes were comparable to those with *Ultraflex*™.

## Discussion

This series shows that CSMS placement alleviates symptoms, restores oral intake, and allows temporary home care in patients with malignant ETBF. Although prognosis remains poor, stenting provides meaningful palliation.

Compared with surgical bypass, CSMS offers equivalent goals—prevention of aspiration pneumonia and restoration of oral intake—through a minimally invasive method suitable for terminally ill patients.^13^ Flexible bronchoscope-guided CSMS insertion requires only local anesthesia and mild sedation, unlike silicone stent placement, which demands rigid bronchoscopy, specialized instruments, and general anesthesia. Double stenting provides more reliable fistula closure and can relieve both esophageal and airway stenosis.^10–12,14,15^ However, in ETBF without concomitant stenosis, a single stent may suffice. Importantly, airway stents, especially CSMS, carry risks of sputum obstruction, migration, or necrosis-induced fistula enlargement; their use should be carefully considered.

Case 3 suggests that esophageal stenting alone can adequately control ETBF in selected patients. While esophageal stenting alone may alleviate fistula-related symptoms, tumor expansion induced by stent deployment can compress the airway and cause respiratory distress.^14^ Nomori et al. recommended that the tracheobronchial stent be inserted first to ensure that the airway remains open, as insertion of the esophageal stent first during “double stenting “might cause tracheobronchial obstruction depending on the location of the esophageal stenosis.^15^

Among these seven patients, the patient (case 7) in whom the bronchial stent insertion was combined with a gastrostomy had the longest survival. Although it’s difficult to make a general statement about the benefit of this treatment due to the different stages of disease progression in the cases, the fact that sufficient nutritional support could be maintained through the gastrostomy, as compared with oral intake, may have contributed to the relatively good outcome in this patient.

Malnutrition is a well-recognized adverse prognostic factor in patients with advanced esophageal cancer,^16^ and has been shown to negatively impact survival outcomes.^17^ However, in this clinical setting, nutritional improvement is often difficult to achieve, and there is currently no robust evidence that nutritional interventions directly prolong survival.^18^ Nevertheless, nutritional support remains important, as it may alleviate treatment-related toxicity, preserve functional status, and contribute to maintaining or improving QOL, even if a direct survival benefit has not been demonstrated.^19^

Limitations: This was a retrospective single-center study with a small sample size. QOL was not systematically assessed. Nonetheless, our findings emphasize that CSMS treatment allows patients with malignant ETBF to spend more of their remaining life at home.

## Conclusion

CSMS placement is a safe, minimally invasive, and effective palliative strategy for malignant ETBF. Double stenting enhances fistula closure when both esophageal and airway stenosis are present, while single stenting may be sufficient otherwise. This approach restores oral intake, prevents aspiration, and supports home-based care in terminally ill patients.

## Authors’ Contributions

K.E., T.N., and T.T. contributed to treatment of the patients. All authors have read and approved this article.
